# The predictive function of Swedish word accents

**DOI:** 10.3389/fpsyg.2022.910787

**Published:** 2022-07-28

**Authors:** Mikael Roll

**Affiliations:** Centre for Languages and Literature, Lund University, Lund, Sweden

**Keywords:** phonology, prediction, prosody, morphology, speech processing

## Abstract

Swedish lexical word accents have been repeatedly said to have a low functional load. Even so, the language has kept these tones ever since they emerged probably over a thousand years ago. This article proposes that the primary function of word accents is for listeners to be able to predict upcoming morphological structures and narrow down the lexical competition rather than being lexically distinctive. Psycho- and neurophysiological evidence for the predictive function of word accents is discussed. A novel analysis displays that word accents have a facilitative role in word processing. Specifically, a correlation is revealed between how much incorrect word accents hinder listeners’ processing and how much they reduce response times when correct. Finally, a dual-route model of the predictive use of word accents with distinct neural substrates is put forth.

## Introduction

Swedish words are lexically associated with tonal *word accents* (Elert, 1964). However, the word accent contrast has a questionable phonological function. From a traditional contrastive perspective (Trubetzkoy, 1958), the word accent distinction is often said to have a low *functional load* (Elert, 1972; Riad, 2014; Althaus et al., 2021). Specifically, in Swedish, although word accents are in principle lexically distinctive, in practice, they do not have any relevant role in distinguishing words from each other. The number of minimal pairs is only in the order of a few hundred. Elert (1972) presented a list of 357 minimal pairs, but noted that many were based on archaic word forms, like the 2nd person imperative accent-2 word ^2^*träden* “step!/thread!” contrasting with accent-1 ^1^*träden* “the trees.” Further, as the previous example illustrates, distinctive pairs often involve different word classes. Their members are hence unlikely to occur in the same syntactic context. Lastly, even the few within-word-class contrasts are questionable as minimal pairs in the traditional sense since their morphological structure differs consistently (Riad, 2014). Typically, the accent-1 words have monosyllabic stems (^1^*and-en* “the duck”), whereas the accent-2 words have disyllabic stems (^2^*ande-n*), involving a stem vowel like *-e* (Riad, 2015).

In Norwegian, there is a much higher number of minimal pairs: at least 2,432 (Jensen, 1958) and possibly 3,000 or more, depending on the criteria used (Leira, 1998). It is thus easy to agree with the view that Swedish word accents have a low functional load. However, since lexical word accents are thought to have been in use already in Late Proto Norse, somewhere between the years 600 and 800 (Riad, 1998), an inevitable question arises. Why has the language kept this apparently useless distinction for over a thousand years and shows no signs of losing it? There does not seem to have been any previous stage with a higher functional load of word accents. On the contrary, the larger extension of the contrast in Norwegian is mainly due to a diachronically fairly late change of unstressed vowels into /e/ and a general reduction of unstressed syllables to [ə], making previously different forms become segmental homophones (Elert, 1964, 1981). It is hence not Swedish that has lost contrasts, but Norwegian that has gained them (Riad, 1998).

## Swedish word accents

**FIGURE 1:**
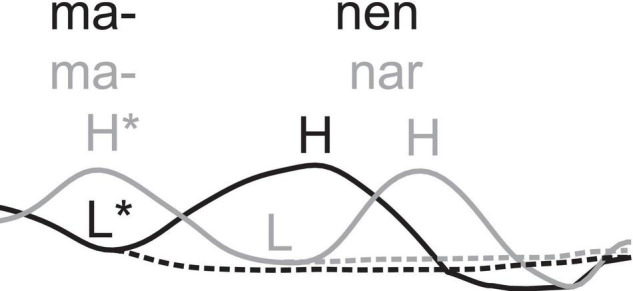
Pitch contour of the accent-1 (L*H) word *manen* “the mane” (black lines) and the accent-2 (H*LH) word *manar* “manes” (gray lines). Solid lines represent focused realizations. Dashed lines show unfocused realizations, L* for accent 1 and H*L for accent 2.

Swedish word accents consist of two distinct word melodies, *accent 1* and *accent 2* (Elert, 1964; Figure 1). Accent 1 is often assumed to be the default intonation of a stressed syllable in the absence of a lexical specification (Riad, 2014). In Central Swedish, it is realized as a low tone associated with the stressed syllable of a word (L*). If the word is in a semantically focused context, a rise to a focal high (H) tone is added, giving L*H (Figure 1, example 1a). Accent 2 can be assigned lexically or post-lexically. Post-lexical accent 2 is found in all words with secondary stress, involving compounds like ^2^ˈ*lejon*ˌ*man* “lion’s mane” and words with stressed suffixes, such as the derivational suffix *-*ˌ*het* “-ness” in ^2^ˈ*när*ˌ*het* “closeness.” The secondary stressed syllable, *man* “mane” in example (1b) has a pattern similar to that of the stressed syllable of accent 1 (1a): a L*, which can be followed by a focal H, yielding L*H. Specifically for accent 2, however, the primary stressed syllable—the *lej* of *lejon* “lion” in example (1b)—has a H*L pattern, producing a two-peaked H*L*H pitch contour in focused words. Lexically assigned accent 2 is phonetically similar to post-lexical accent 2, but occurs in words without secondary stress, such as *manar* “manes” (1c). It is also pronounced as a high tone followed by a fall in the stressed syllable (H*L). Since the only stressed syllable is already associated with a non-focal H*, the focal H is realized in the posttonic syllable, producing a two-peaked H*LH sequence in focused words but without secondary stress (Riad, 1998).

**Table:** 

(1)		
a. *Accent 1*	b. *Post-lexical accent 2*	c. *Lexical accent 2*
L*H	H*L L*H	H*L H
ˈman -en	ˈlejon -ˌman	ˈman-ar
mane -DEF.SG	lion mane	mane-PL

“Lexically” assigned does not mean that the word accent is marked for lexemes. Instead, it is conditioned by the word’s morphology (Rischel, 1963). Although word accents are realized on the stressed stem syllable, lexical accent 2 is specified for the stem by a specific set of unstressed suffixes such as *-ar* “-PL (2nd declension)” and *-te/de* “-PST (2nd conjugation),” or stem vowels, as the *-e* of *ande* “spirit.” In contrast, accent 1 is a post-lexical realization of a prosodic word not involving any accent 2-inducing morpheme or secondary stress (Riad, 2012). All monosyllabic words have accent 1, but there are also many suffixes that are unmarked for word accent, like *-(e)n* “SG.DEF (2nd declension)” and *-(e)r* “PRS (2nd conjugation),” and therefore occur in words with the default accent 1. In this view, word accents are almost entirely redundant, derivable from stress patterns and suffix information, if not affected by additional phonological processes altering the specified accent (Elert, 1972; Myrberg and Riad, 2015). The accents’ redundancy explains their low functional load in the traditional sense; word accents are not used for lexical contrasts but are rather a morphological bi-product. However, whereas post-lexical word accents follow transparent rules, the set of suffixes that triggers accent 2 seems more arbitrary from a synchronic perspective. The next section provides an account for the origin of lexical word accents (Riad, 1998), and sharpens the question of why they have been conserved.

## The rise of lexical word accents

Several hypotheses have been advanced about the origin of Scandinavian lexical word accents (Kock, 1878; Öhman, 1967). Riad (1998) particularly well explained the relation between post-lexical and lexical accents and the morphological conditioning of lexical accent 2. Simply put, Riad (1998) derived lexical accent 2 from the still present post-lexical accent 2. His explanation built on the observation that accent 2 without secondary stress is mainly found in words with suffixes that are likely to have been stressed in Early Proto Norse. For example, the Modern Swedish accent-2 word *satte* “put.PRT” contains the past tense suffix -de/-te. All words with this suffix have accent 2, like ^2^*följ-de* “followed” and ^2^*köp-te* “bought.” Nonetheless, as explained in the previous section, their stems can have accent 1 if combined with an unspecified suffix, such as the present tense conjugation -*er* in ^1^*följ-er* “follows” or the hypocoristic derivational nominalizer *-is* (Riad, 2012) in the neologism ^1^*köp-is* “shopping center” of *Valbo Köpis* “Valbo Shopping Center.” The reconstruction of the suffix corresponding to past tense *-de/-te* in Early Proto Norse is *-*dee* “-3SG.PRT,” as in *ˈsati-ˌ*dee* “put-3SG.PRT,” with primary stress on *sat-* and secondary stress on -*dee*. In focus, this two-stressed pattern would trigger a post-lexical, two-peaked accent-2 pitch pattern in modern Central Swedish. If post-lexical prominence rules were similar in Proto Norse, words like *satidee would hence have two pitch peaks. During the syncope period in Late Proto Norse, many intermediate unstressed syllables disappeared, leaving a large number of word forms like *ˈ*sat*ˌ*tee* “put.3PRT” with two adjacent stressed syllables. This led to stress clash resolution removing the secondary stress, giving the Modern Swedish form ˈ*satte* “put.PRT,” with only one stressed syllable (Riad, 1992). However, while reducing the length and weight of what had been the secondary stressed syllable, the stress clash resolution left the word melody intact, still with two peaks in a focused position. The pitch contour would then have been reinterpreted as being lexically marked for the specific suffixes rather than the result of applying a post-lexical rule (Riad, 1998). This is where the main question of this article takes shape: Why was the pitch pattern kept when its motivating secondary stress disappeared and why has it been conserved as a lexical accent ever since?

## The processing perspective

Elert (1964, 1972, 1981) mentioned two alternative potential functions of word accents besides the lexically contrastive. On the one hand, he argued that one function could be to distinguish different morphemes—chiefly grammatical suffixes—from each other. Thus, whereas the participle suffix *-en* in ^2^*brut-en* “broken” induces accent 2 onto the stem, the singular definite *-(e)n* in ^1^*bil-en* “the car” is unmarked for word accent and, therefore, occurs with accent 1. The accent 2-marking for the preceding syllable is what distinguishes the participle suffix from the singular definite. Another role he attributed to accent 2 is the *connective* function. Accent 2 never occurs in monosyllabic words since it is conditioned by secondary stress, suffixes, or stem vowels occurring in a syllable following the primary stressed syllable. This characteristic makes for a potential function of accent 2 in indicating that a word is necessarily polysyllabic (Elert, 1964). However, the morphological and connective functions are both largely redundant from a systemic point of view. The association of word accent with suffix leads only to a handful of contrasts like ^1^*biten* “the piece” and ^2^*biten* “bitten,” which are included in Elert’s list of minimal pairs. In most cases, neither definite nouns have participle segmental homophones nor participle forms have nominal homophones. There is no *^2^*bilen* or *^1^*bruten* corresponding to ^1^*bilen* “the car” and ^2^*bruten* “broken.” Furthermore, since nouns and participles are used in different syntactic environments, word accents are unlikely ever to be needed to distinguish the morphemes.

Post-lexical accents might have a connective function. Specifically, accent 2 can show that two stressed syllables belong to the same syntactic word,^1^ and thus make a difference between a phrase like ^1^ˈ*fin*^1^ˈ*hatt* “nice hat” and a compound such as ^2^ˈ*fin*ˌ*hatt* “fine hat.” The phrase and the compound are similar in having two stressed syllables, but, in the phrase, both monosyllables have accent 1, whereas the compound has accent 2 due to its secondary-stress pattern. Nevertheless, the connective function is very weak for lexical accent 2. Polysyllabic words with only one stressed syllable can have either accent 1 or 2. Only in a few cases does the word accent actually distinguish between different forms. It happens under particular syntactic conditions when a suffix and an unstressed verb are homophonous (Elert, 1964). In this vein/’rostar/is understood as a disyllabic verb with the accent 2-inducing suffix *-ar* “-PRS” if pronounced with accent 2 as in example (2)a. If the sequence is uttered with accent 1, it will be interpreted as consisting of two words: the name Ross, followed by the verb *tar* “takes,” as indicated in (2)b.

**Table:** 

(2)		
	a.	ˈ^2^rost-ar ˈledningen,
		rust-PRS the.wire
		“Does the wire rust?”

	b.	ˈ^1^Ross tar ˈledningen
		Ross takes the.lead

Even the few cases of this type are problematic as arguments for a connective function of lexical accent 2. The verb would not need to be deaccented in example (2b). If it were not, the stress pattern would also have differed between the two sentences. The same is true for the sentence presented by Elert (1964).^2^ In other words, lexical accent 2 does not seem to have an essential distinctive function in showing that a stressed and an unstressed syllable together form a word.

As we have seen, even word accents’ morphological and connective functions are largely redundant when viewing language statically as a system. They gain a different sense, however, if a dynamic processing approach is taken. The word accent distinction is perceived in the stressed syllable of a word, often word-initially (sometimes perhaps even in the pre-tonic syllable) as a L* or H* tone. At this point of perception, the suffix, stem vowel, or secondary stress that might have induced accent 2 has not yet been perceived. Thus, at the time point when the word accent distinction becomes audible, it offers non-redundant information about the upcoming structure. At this stage, the tone can have more of a distinctive function. To be exact, most psycholinguistic models assume that the initial speech sounds of an unfolding word (pre-)activate the possible words the listener might be perceiving, the *lexical competitors*. The subsequent sounds reduce the lexical competition by inhibiting competitors that are incompatible with the unfolding sequence of speech sounds, narrowing down the selection to a point where there is only one candidate word left (McClelland and Elman, 1986; Marslen-Wilson, 1987; Norris and McQueen, 2008). In this sense, just like the segments, word accents can help the listener determine which word s/he is listening to. If we hear example (3) with a L* accent 1 tone on *ren-* “reindeer-,” we know almost for sure that the noun is definite singular even before hearing the *-en* “-DEF.SG” suffix expressing that information, due to the probabilistic connection between accent 1 and the suffix. If the target word instead involved the accent 2-inducing plural suffix *-ar* “-PL,” as in example (4), the stem *ren-* “reindeer” would be pronounced with a H* accent-2 tone. Again, upon hearing the H* tone on the stem, we would strongly expect the associated suffix *-ar* “-PL” to follow.

**Table:** 

(3)	kälk-en	drogs	av	^1^ren-en
	sledge-DEF.SG	was.pulled	by	reindeer-DEF.SG
	“The sledge was pulled by the reindeer”			

(4)	kälk-en	drogs	av	^2^ren-ar
	sledge-DEF.SG	was.pulled	by	reindeer-PL
	“The sledge was pulled by reindeers”			

## The predictive function

Recent research has highlighted the predictive nature of speech processing (Kuperberg and Jaeger, 2016; Friston et al., 2021). Rather than processing sounds as they arrive, the brain is thought to constantly entertain weighted hypotheses about what it will perceive next. When the auditory evidence arrives, brain areas of lower-level processing pass on information to higher-level areas about what *does not* conform to the hypotheses. The *prediction error* report is used to fine-tune the predictive model to make predictions even better in the future. Since the major part of our perceptual environment is relatively stable, this *predictive coding* is energetically more cost-effective than treating all information as unexpected (Friston, 2009). It is against this backdrop that I argue that the chief function of word accents and the explanation for their millenary survival is to be found. Word accents are good predictors of how words will continue during processing, and their primary function is predictive. Their role in prediction can be related to their morphological and connective functions. From a processing perspective, word accents can have a quasi-distinctive status as cues to their associated upcoming suffixes. Accent 2 is also a cue to a possible upcoming secondary stress.

There is a relatively large body of evidence that word accents influence prediction. Firstly, if they are combined with the wrong suffix, it takes a longer time to respond to the grammatical meaning conveyed by the suffix (Söderström et al., 2012; Roll et al., 2013, 2015; Roll, 2015; Novén, 2021). For example, if listeners hear *ren-* “reindeer” with accent 1 L* and then the word continues with the accent 2-associated suffix -*ar* “-PL,” it takes them longer to decide whether the word is singular or plural than if the correct word accent-suffix combination would have been delivered. Secondly, the surprise at a suffix that is unexpected due to the word accent can also be seen in a brain potential called *P600* (Roll et al., 2013, 2015; Roll, 2015; Novén, 2021). The P600 is an electrically positive brain wave typically peaking at 600 ms following syntactically (Osterhout and Holcomb, 1992) or morphologically (Rodriguez-Fornells et al., 2001) unexpected forms. It has been argued to index reanalysis of the unexpected structure (Morris and Holcomb, 2005).

The fact that word accents *can* be used predictively when relevant to the task (judging suffix-based meaning) does not necessarily entail that they have a predictive role in other contexts. However, even using an acceptability judgment task, Roll et al. (2010) observed a P600 effect for incorrect combinations of word accent and suffix. The experiment additionally involved declensionally incorrect words like *minkor “minks,” where the 1st-declension plural *-or* suffix has replaced the correct 2nd-declension plural *-ar* of *minkar* “minks.” Although both suffixes induce accent 2, only -*ar* is of the right declension class. Acceptability was only slightly affected by incorrect combinations of word accent and suffix but was mainly based on the correctness of the declension and the semantic characteristics of the sentences. This implies that the association between word accent and suffix was not perceived as particularly relevant for the task. Likewise, in a study with a task where participants pressed a button at the sentence boundary, suffixes that were invalidly cued by the wrong word accent also produced an increased P600 (Gosselke Berthelsen et al., 2018). In sum, the surprise effect when hearing an incorrectly cued suffix seems relatively task-independent.

Accent 1 is generally a better predictor than accent 2. The reason is that accent 1 reduces the lexical competition more at the point where the stressed syllable is perceived (Söderström et al., 2016). When hearing it, the listener can inhibit the wide range of hypotheses of upcoming possibilities associated with accent 2. Since there are fewer possible continuations, the prediction is more certain when a stem has accent 1. Accent 1 is, to put it in another way, more constraining in processing. As mentioned above, all prosodic words with secondary stress are assigned accent 2 post-lexically. Since compounds have secondary stress, all compounds consequently have accent 2. There are also more inflectional (Riad, 1998) and derivational (Riad, 2012) suffixes that are marked for accent 2 than there are unmarked suffixes. In fact, in a corpus, word-initial syllables with accent 2 had 10.5 times as many possible continuations (10.5 times higher lexical competition) as word-initial syllables with accent 1 (Söderström et al., 2016). The difference in the certainty the two word accents entail can be illustrated by examples (3) and (4). Whereas ^1^*ren*- with accent 1 has only one possible continuation, ^2^*ren-* with accent 2 has several, for instance, *spannet* “the team,” giving the accent-2 compound *renspannet* “the reindeer team.” Hence, even if plural *-ar* is the most likely continuation, the listener cannot be as confident upon hearing the accent-2 stem as when hearing the accent-1 stem. The constraining effect of accent 1 is evidenced by listeners’ increased surprise when it is invalidly followed by accent 2-inducing suffixes. The P600 has been found to be larger for invalidly cued accent 2-inducing suffixes, indicating greater morphological reanalysis effects (Roll et al., 2010, 2013). Response times have also been relatively longer for accent-2 suffixes incorrectly preceded by an accent 1 tone on the stem than for unmarked suffixes invalidly cued by accent 2 (Söderström et al., 2012; Roll, 2015).

The higher certainty led to an increase for accent 1 in another brain potential already when participants heard the pitch onset of the word-initial syllable: the *pre-activation negativity* (PrAN) (Roll, 2015; Roll et al., 2015; Söderström et al., 2016, 2017). The PrAN has been seen to be greater the more predictively beneficial a speech sound is (Roll et al., 2017), in both suffix meaning-based tasks and acceptability judgment tasks (Söderström et al., 2016). The PrAN effect of accent 1 was absent in early second language learners, who still had not acquired the predictive use of word accents (Gosselke Berthelsen et al., 2018). However, after intense training, this electrically negative brain potential increased for both word accents, but significantly more for accent 1 (Hed et al., 2019). The results indicate that second-language learners acquired a general predictive use of word accents and learned that accent 1 is a better predictor than accent 2. In short, Swedish-speaking listeners can use word accents predictively during active listening and not only when it is beneficial for a particular task.

Presenting the predictive function in terms of which suffixes word accents pre-activate is overly simplistic. Word accents can often reduce the lexical competition before the listener even knows which stem s/he is perceiving. Already when the initial two segments of a word become apparent, an intense reduction of the available lexical candidates can occur (Marslen-Wilson, 1987; Roll et al., 2017). We cannot directly measure this lexical selection as we cannot access each Swedish speaker’s mental lexicon. Still, we can estimate a possible mental lexicon by combining a large speech corpus with a pronunciation lexicon (Söderström et al., 2016). An average speaker can be assumed to have been exposed to words with the approximate frequency and distribution in a corpus with sources representing different language registers. Relating the corpus^3^ to the pronunciation dictionary (Andersen, 2011) makes it possible to extract the number of words that begin with a particular sequence of phonemes and their relative frequency.

Taking as an example the last word of (3)–(4), we find that 4,261 nouns begin with /r/. Hearing a following /e/ reduces the lexical competitors to 305 candidates, 7.2% of the initial number. If word-accent information is added, even more substantial inhibition of candidates is achieved. Accent 2 lowers the number to 286, whereas Accent 1 decreases the quantity to 19 possibilities. Hence, when perceiving the second segmental phoneme of the word, accent 1 offers an additional 93.8% reduction of the lexical competitors, whereas accent 2 cuts the number by 6.2%. The example illustrates the general tendency for accent 1 to drop lexical candidates to a much greater extent than accent 2. If we inspect the competitors supported by each word accent, we can see that this quantitative generalization is related to the connective and morphological functions of accent 2, but cannot be reduced to them. The accent-2 group contains words with secondary stress (e.g., *researrangören* “the tour operator” and *renhet* “purity”), words derived by specified suffixes like *-are* [e.g., *redare* “ship-owner(s)”] or stem vowels (e.g., the second *e* in *redet* “the nest”), in addition to the plural *-ar* inflection already mentioned (e.g., *renar* “reindeers”). Although the variation among the accent-1 competitors is much more limited, not only singular suffixes, such as the already mentioned singular definite -*(e)n* of the 2nd-declension word (*renen* “the reindeer”) and 5th-declension *-(e)t* of *repet* “the rope,” appear, but also the 5th-declension plural inflection *-(e)n* in *repen*, which is also unmarked for accent 2. This illustrates the fact that accent 1 drastically limits the number of morphological possibilities but does not exclude all of them. It should be mentioned that, above, I have disregarded semantic factors that are also liable to play a role in constraining the likelihood of different lexical competitors (Marslen-Wilson, 1987). This section has shown that the pre-activation cued by word accents can be assumed to precede the recognition of the full stressed syllable and to consist to a large extent of suppression of irrelevant alternatives.

## The facilitative function

It seems likely that Swedish speakers use word accents to predict upcoming morphemes and word structure and narrow down the lexical competition during listening. Nevertheless, the results reviewed so far do not show that word accents actually facilitate processing. They confirm that incorrect word accents hinder the processing of suffixes, producing a *retardation effect*—slower response times for suffix-based judgments. Incorrect combinations of word accent and suffix also call for reanalysis of the word’s morphological structure, as seen in the P600 brain potential. Nonetheless, these effects do not show that the tones make processing faster when they are correct. A *facilitative* role in this sense would be necessary to argue that the predictive function of word accents has been decisive for their survival. This indeed finds support in correlations observed between brain structure measures and skills in the native language. Specifically, a correlation has been detected between the cortical thickness of areas related to phonological and word form processing, involving Wernicke’s area, and how much the suffix processing is slowed down by invalid word accents (Schremm et al., 2018; Novén et al., 2021). The participants with thicker cortex in Wernicke’s area also showed faster response times for suffix-based meaning in words with correct combinations of word accent and suffix (Schremm et al., 2018). The fact that a thicker cortex in Wernicke’s area is related to both quicker processing of valid connections between word accent and suffix and increased impediment in handling invalid combinations suggests that enhanced predictive use of word accents indeed implies better performance in terms of rapid processing of words. Even so, for the purpose of establishing a relation between the processing speed of words and the predictive use of word accents, the link involving the cortex is indirect. A direct relation between the use of word accents and word-processing speed has never been tested.

We can formulate a hypothesis for the facilitative function in the following way: If word accents have a facilitative role, a person who gives them more weight during processing should also process words faster than someone who gives less weight to the word accent information. The facilitative hypothesis can be tested using previously collected response time data from Central Swedish (Roll et al., 2015) and South Swedish (Roll, 2015). I will soon return to the empirical support for the hypothesis but will first present the retardation effect, which is necessary to appreciate the evidence. In Roll et al. (2015) and Roll (2015), participants listened to definite singular or indefinite plural nouns presented in short carrier sentences, for example, *hatt-en* “the hat” or *hatt-ar* “hats” in *Kurt fick hatten/hattar till jul* “Kurt got the hat/hats for Christmas.” The same sentences were recorded in the two dialects.^4^ Thirty different nouns were presented in singular definite and plural indefinite forms. Half of the stimuli were spliced to create invalid combinations of the word accent realized on the stem and the following suffix.^5^ For example, *hatt* “hat” was presented in the valid forms ^1^*hatt-en* “the hat” and ^2^*hatt-ar* “hats,” and in the invalid forms ^*2^*hatt-en* “the hat” and ^*1^*hatt-ar* “hats.” The task was to judge, as quickly as possible, whether the word was singular (*en* “one”) or plural (*flera* “several”). To put it differently, the participants judged the suffix-based part of the meaning of the target words.

**FIGURE 2:**
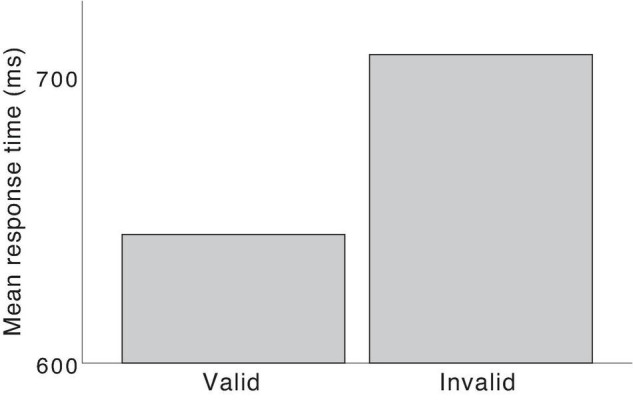
Retardation effect. Suffixes in words with invalid combinations of word accent and suffix had longer response times than suffixes in words with valid combinations, *t*(79) = 5.93, *p* < 0.001.

If word accents are used predictively, the listeners would be thought to create an expectation for the suffix already when hearing the word stem. If they heard a stem with accent 1, they should predict an upcoming *-en* “-SG.DEF” suffix. If they perceived an accent-2 stem, they would expect a following *-ar* “-PL.” The listeners’ expectation should lead to increased response times if an unexpected suffix was delivered due to invalid combinations of stem tone and suffix. As mentioned in the previous section, this retardation effect on suffix processing for invalid word accents has been extensively shown. The retardation is the increased response time for suffixes that have been invalidly cued by a stem with the wrong word accent compared to the same suffixes when validly cued by a stem with the correct word accent (Söderström et al., 2012, 2017; Roll et al., 2013, 2015; Roll, 2015). Figure 2 shows the retardation effect for the joint data for nouns in Roll et al. (2015) and Roll (2015). However, the retardation effect *per se* does not tell whether the word accents have a facilitative function in valid words.

**FIGURE 3:**
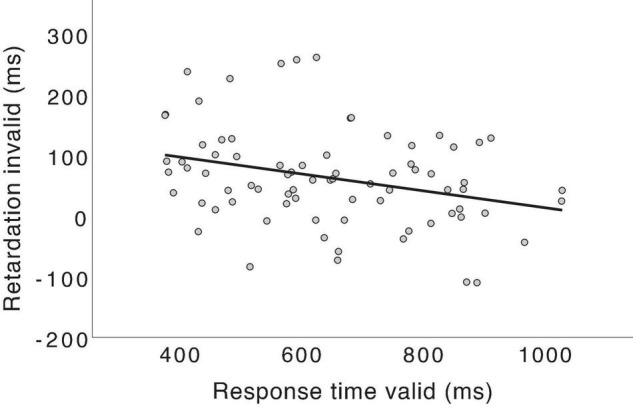
Response time for valid words was the best predictor of the retardation effect for invalid words. A regression line with response time in valid words as the only predictor of the retardation effect is shown, *R*^2^ = 0.093, *F*_(1, 75)_ = 7.69, *p* = 0.007.

In order to test the facilitative hypothesis, we will now reanalyze the previous response time data to assess whether valid word accents improve processing speed. The reasoning is as follows. If an individual relies more on word accents in his/her processing of suffixes than others, that person should show a greater retardation effect for invalid word accents. Further, if word accents have a facilitative effect, the person depending more on the pitch information would benefit more from hearing valid word accents than others who do not rely as much on the pitch. Therefore, s/he should also be faster than others in processing suffixes of valid words. This will show in a regression model, where an individual participant’s response times for valid words should predict the same individual’s retardation effect for invalid words. I have tested the hypothesis using linear regression in SPSS (IBM Corp, 2021) on the data in Roll et al. (2015) and Roll (2015) with the retardation effect of invalid word accents as the dependent variable. The retardation effect was calculated as the subtraction of each participant’s average response time for a suffix that was validly cued by the correct word accent from the response time for the same suffix when invalidly cued by the incorrect word accent. The response time for validly cued suffixes was entered as an independent variable. Other variables that might explain the retardation effect were also included: word accent and dialect. These variables were dummy coded with values 0 for accent 1 and 1 for accent 2, as well as 0 for Central Swedish, and 1 for South Swedish. Outliers of more than 3 standard deviations over or under the average of each variable were removed. The model was significant, *R*^2^ = 0.251, *F*_(3, 73)_ = 8.15, *p* < 0.001, explaining 25.1% of the variance in the data. The response time in valid words was the strongest predictor of the retardation effect (standardized β = –0.352, *p* = 0.001) (Figure 3), but word accent (standardized β = –0.292, *p* = 0.005) and dialect (standardized β = 0.270, *p* = 0.011), were also significant predictors. To make sure that retardation was specifically related to faster response times for valid words, I also ran the same regression model but included response time for invalid words as an independent variable instead of the response time for valid words. Invalid-word response time did not predict retardation (standardized β = 0.064, *p* = 0.579).

The regression results show that persons who relied more on word accents during processing also specifically processed valid words faster. In other words, word accents indeed had a facilitative effect. There was also a difference between accents 1 and 2 pointing in the same direction as has previously been found: accent 1 in the stem has a greater retardation effect. As mentioned above, accent 1 is a stronger predictor due to its occurrence in fewer possible words (Roll, 2015; Roll et al., 2015; Söderström et al., 2016). Therefore, it generates stronger activation of its compatible lexical competitors and inhibition of the incompatible candidates, leading to enlarged prediction error and retardation for failed predictions. I will refrain from interpreting the difference between dialects since the speech rate and the focus patterns of the stimuli were not controlled between the two experiments. However, it is worth mentioning that a slightly weaker connective function of accent 2 in South Swedish due to accent 1 also occurring in some compounds (Bruce, 1973; Frid, 2000; Riad, 2015) has been argued to give rise to a somewhat smaller difference in the predictive power of accent 1 and 2 (Roll, 2015).

## Dual-route prediction

It now seems clear that word accents have a facilitative function as cues to predict upcoming morphemes and word structure. This implies that Swedish speakers have learned and stored links between tones on stems and specific suffixes. They can further be thought to have associations between an accent-2 tone and an upcoming secondary-stressed syllable. Nevertheless, it is not self-evident how the brain stores the connections between tone and suffix from which the predictions emanate. Based on the dual-route model of morphological processing (Pinker, 1991), there are two chief alternatives for the association between tone and suffix. On the one hand, there can be a more abstract, rule-like connection, something like H*-ar, intending to say that all -*ar* “-PL (2nd declension)” suffixes must be preceded by a H* (accent 2) in the stressed syllable. There is also the possibility that words are stored as fully inflected forms, together with their word accent, in representations like ^H*^bilar “cars” and ^H*^manar “manes,” etc. It is easy to tell that the rule-like option is more parsimonious. Only one association is needed for all words involving the 2nd-declension plural suffix. At the same time, the full-form type storage can be thought to allow for quicker lexical access. When hearing ^H*^man… a listener would immediately activate the full word ^H*^manar “manes” as the most likely option without having to go through a compositional process where, upon hearing the stem, a suffix is selected based on the knowledge about the possible declension and the tone. This option is also more in line with the lexical competition models presented above.

The most apparent evidence of the existence of an abstract association between word accent and suffix comes from a paradigm where pseudoword stems were combined with real suffixes, giving words like *kvup-en* “kvup-SG.DEF” or *kvup-ar* “kvup-PL.” As in the experiments reanalyzed in the previous section, the task was to judge the suffix-based meaning, whether the word was in singular or plural form. Sometimes the suffixes were masked by a cough, leaving the word accent as the only cue to the number. Still, it was relatively easy for the participants to perform the task even without hearing the suffixes. The accuracy was as high as 88% for accent 1 and 72% for accent 2 (Söderström et al., 2017), indicating that the participants activated the suffix based only on the word accent since the pseudowords used cannot have had any full-form storage. The lower performance for accent 2 is natural because the words, although less likely, could have been singular compounds, all compounds involving accent 2, or could have had a disyllabic stem with an accent 2-inducing stem vowel like *-e* in *kvup-e*. The word accents were also used predictively. As in real words, invalid combinations of word accents and suffixes led to longer response times and P600 effects for the invalidly cued suffixes. A P600 increase has likewise been observed for invalid combinations of accent 1 with accent 2-inducing suffixes, even if the suffixes were declensionally incorrect, as the *^1^mink-or “mink-PL (2nd declension stem-1st declension suffix)” mentioned above (Roll et al., 2010). Furthermore, accent 1 in pseudowords also produced an increased PrAN compared to accent 2 (Söderström et al., 2017). This is because, as mentioned above, the post-lexical accent-2 rule for secondary stressed words applies even in pseudowords, meaning that accent-2 stems yield more possibilities and thus lower certainty. In brief, the word accent-based prediction can proceed combinatorially. Signs of combinatorial processing have also been found for the interaction of stress with suffix in Swedish (Zora et al., 2019). However, it is not evident that this is the preferred route for real nouns (Lehtonen et al., 2009; Schremm et al., 2019).

It has been proposed that word accents of frequent real nouns are stored together with full inflected word forms for quick access (Schremm et al., 2018). Accordingly, as mentioned already, the cortical thickness of Wernicke’s area and other temporal brain areas correlated with greater predictive use of word accents in real words (Schremm et al., 2018; Novén et al., 2021). Nonetheless, the same kind of increase in response time for invalid combinations of word accent and suffix in pseudowords did not correlate with cortical thickness in those areas. Instead, there was a correlation with the cortical thickness in Broca’s area in the left frontal lobe (Schremm et al., 2018). Broca’s area is known for its involvement in combinatorial processing (Ullman et al., 1997). Therefore, Schremm et al. (2018) interpreted the results as showing different neural substrates for the capacity to use word accents predictively in combinatorial and full form-based processing. The brain areas are in line with recent neurolinguistic models situating word processing mainly in the temporal lobe (DeWitt and Rauschecker, 2012) and combinatorial processing in Broca’s area (Friederici et al., 2017).

## Discussion

The article has asked what the primary function of Swedish word accents is. Lexical word accents have existed in Swedish for probably over a thousand years. Yet, word accents are not really used to distinguish words and hence have a very low functional load in the traditional phonological sense. There is, however, a large body of psycho- and neurophysiological evidence for possible predictive use of word accents. Due to a strong association between the word accents and suffixes, a listener can use the pitch pattern on a stressed word stem to infer properties in the continued speech string. Elert (1964) argued that word accents have a *morphological* function in distinguishing different suffixes. The morphological function can be said to gain relevance when language is viewed from a dynamic processing perspective rather than as a static system. In this sense, the word accent has a quasi-distinctive function at a point in time before the suffix is perceived. At that point, it can be used to predict the suffix. It might be speculated that predicting words’ suffixes is crucial in a language where definiteness and number are otherwise mainly expressed in the suffix. Many other languages, involving English, German, and Spanish, express definiteness and number in a pre-nominal article, making the information available before hearing the lexical noun. A preposed definite article is also used in Swedish, but only in complex noun phrases, where no information about the definiteness and number would otherwise be inferable at the phrase onset, being outside the scope of the head noun’s word accent pattern. For example, a phrase involving an adjective has an additional initial article doubling the suffix’s definiteness and number, as in *den röda boll-en* “SG.DEF red ball-SG.DEF.”^6^ In these cases, the word accent of the head noun is not perceived at the beginning of the noun phrase, meaning that without the double definiteness marking, information about number and definiteness would only be available upon hearing the noun.

For the first time, this article has shown that the well-known predictive function of word accents, in fact, also involves facilitating word processing. It was revealed that the more listeners relied on word accents in their processing, the faster they processed inflected words with correct word accents. Reliance on word accents was operationalized as the relative increase in response time when judging the meaning of suffixes preceded by the wrong word accent (retardation effect). Finally, the brain can put the predictive function of word accents into practice through two routes with different neural substrates: the combinatorial and holistic routes. In frequent words, word accents seem to be stored and accessed holistically together with fully inflected forms. In essence, upon hearing a stem with a word accent, the listeners activate the linked suffix as part of a word form that is stored with both inflection and word accent as part of the representation. However, the relation between word accent and suffix can also be combinatorially assembled during listening. The combinatorial processing route is probably always activated to some degree, but it is vital in unknown words. More precisely, when hearing an unknown stem with a word accent, the associated suffix is activated through something similar to a grammatical rule, an abstract association between word accent and suffix.

Word accents predict not only suffixes. Since post-lexical accent 2 is used for words with secondary stress, including all compounds, accent-2 stems activate a much larger number of possible continuations. This can be said to be the *connective* function of accent 2 (Elert, 1964) viewed from a speech-processing perspective. Stems with accent 1 can usually only have a limited set of suffixes. Due to the lower number of possible continuations, accent 1 increases the certainty about the continuation of the speech signal and is, therefore, a stronger predictor than accent 2 during listening. In psycholinguistic terms, the pitch-induced certainty is due to a suppression of the lexical competitors that are incompatible with the incoming information. This lexical selection process is likely to gain momentum before the full syllable is recognized, around the point where the first two segmental phonemes become discernable. The higher confidence is indexed by an augmented brain potential, the pre-activation negativity (PrAN), for stems with accent 1. The neural mechanisms underlying the pre-activation of upcoming speech in perception are still being investigated. At present, we do not know to what extent the more prominent neural activity for accent 1 is due to pre-activation of the few alternatives it cues or inhibition of the large number of possibilities associated with accent 2. The most likely scenario is that both processes are involved. Pre-activation can be regarded as a reweighting of hypotheses about the immediate future, strengthening the cued alternatives but inhibiting the uncued. Whether word accents have a low functional load depends on how their function is defined. Here, it is argued that their function is predictive and that they play an essential role in facilitating word processing.

## Data availability statement

The original contributions presented in the study are included in the article/Supplementary material. Further inquiries can be directed to the corresponding author.

## Ethics statement

The studies involving human participants were reviewed and approved by the Lund Local Ethical Review Board. The patients/participants provided their written informed consent to participate in this study.

## Author contributions

The author confirms being the sole contributor of this work and has approved it for publication.

## Footnotes

1 Phonologically, it shows that the two syllables belong to the same maximal prosodic word (Myrberg and Riad, 2015).

2 Elert’s (1964) example was *vår* ‘^2^*svenska flagga* “our Swedish flag” vs. *vår* ’^1^*sven ska flagga* “our swain shall flag.”

3
https://spraakbanken.gu.se/swe/resurs/parole

4 In the Central Swedish stimuli, focus was on the adverbial phrase following the nouns (*till jul* in the example) to avoid having focus on the target word. Focus would create an imbalance between accent 1 and accent 2, since the focal H occurs in the first syllable in accent 1 but in the second syllable in accent 2.

5 Recordings were carefully carried out to avoid pre- and posttonic differences in F0 between accents 1 and 2. Any potential remaining pre-tonic effects were excluded by the balanced insertion of the target words in carrier sentences originally recorded with accent-1 or accent-2 words. The stressed syllable was always surrounded by voiceless segments to improve the splicing. The resulting stimuli sounded like sentences including a word with correct or incorrect word accent, but otherwise natural pronunciation.

6 I thank reviewer 2 for drawing my attention to this fact.

## References

[B1] Althaus N. Wetterlin A. Lahiri A. (2021). Features of low functional load in mono- and bilinguals’ lexical access: evidence from Swedish tonal accent. *Phonetica* 78 175–199. 10.1515/phon-2021-2002 33979904

[B2] Andersen G. (2011). *Leksikalsk Database for Svensk.* Oslo: Nasjonalbiblioteket.

[B3] Bruce G. (1973). *Tonal Accent Rules for Compound Stressed Words in the Malmö dialect”, Working Papers.* Lund: Phonetics Laboratory. Lund University.

[B4] DeWitt I. Rauschecker J. P. (2012). Phoneme and word recognition in the auditory ventral stream. *Proc. Natl. Acad. Sci. U. S. A.* 109 E505–E514. 10.1073/pnas.1113427109 22308358 PMC3286918

[B5] Elert C.-C. (1964). *Phonologic Studies of Quantity in Swedish Based on Material from Stockholm Speakers.* Stockholm: Almqvist & Wiksell.

[B6] Elert C.-C. (1972). “Tonality in Swedish: rules and a list of minimal pairs,” in *Studies for Einar Haugen*, eds Firchow K. G. E. S. Hasselma N. O’Neil W. (The Hague: Mouton).

[B7] Elert C.-C. (1981). *Ljud och Ord i Svenskan 2.* Stockholm: Almqvist & Wiksell International.

[B8] Frid J. (2000). Compound accent patterns in some dialects of Southern Swedish. *Proc. Fonetik* 2000 61–64.

[B9] Friederici A. D. Chomsky N. Berwick R. C. Moro A. Bolhuis J. J. (2017). Language, mind and brain. *Nat. Hum. Behav.* 1 713–722. 10.1038/s41562-017-0184-4 31024099

[B10] Friston K. (2009). The free-energy principle: a rough guide to the brain? *Trends Cogn. Sci.* 13 293–301. 10.1016/j.tics.2009.04.005 19559644

[B11] Friston K. J. Sajid N. Quiroga-Martinez D. R. Parr T. Price C. J. Holmes E. (2021). Active listening. *Hear. Res.* 399:107998. 10.1016/j.heares.2020.107998 32732017 PMC7812378

[B12] Gosselke Berthelsen S. Horne M. Brännström K. J. Shtyrov Y. Roll M. (2018). Neural processing of morphosyntactic tonal cues in second-language learners. *J. Neurolinguistics* 45 60–78. 10.1016/j.jneuroling.2017.09.001

[B13] Hed A. Schremm A. Horne M. Roll M. (2019). Neural correlates of second language acquisition of tone-grammar associations. *Ment. Lex.* 14 98–123. 10.1075/ml.17018.hed 33486653

[B14] IBM Corp (2021). *IBM SPSS Statistics for Macintosh*, 28 Edn. Armonk, NY: IBM Corp.

[B15] Jensen M. K. (1958). *Bokmålets Tonelagspar (”Vippere”).* Bergen: A.S. John Griegs Boktrykkeri.

[B16] Kock A. (1878). *Språkhistoriska Undersökningar om Svensk Akcent.* Lund: Gleerup.

[B17] Kuperberg G. R. Jaeger T. F. (2016). What do we mean by prediction in language comprehension? *Lang. Cogn. Neurosci.* 31 32–59. 10.1080/23273798.2015.1102299 27135040 PMC4850025

[B18] Lehtonen M. Vorobyev V. Soveri A. Hugdahl K. Tuokkola T. Laine M. (2009). Language-specific activations in the brain: evidence from inflectional processing in bilinguals. *J. Neurolinguistics* 22 495–513. 10.1016/j.jneuroling.2009.05.001

[B19] Leira V. (1998). Tonempar i bokmål. *NOR-skrift* 95 49–86.

[B20] Marslen-Wilson W. D. (1987). Functional parallelism in spoken word-recognition. *Cognition* 25 71–102. 10.1016/0010-0277(87)90005-9 3581730

[B21] McClelland J. L. Elman J. L. (1986). The TRACE model of speech perception. *Cogn. Psychol.* 18 1–86. 3753912 10.1016/0010-0285(86)90015-0

[B22] Morris J. Holcomb P. J. (2005). Event-related potentials to violations of inflectional verb morphology in English. *Cogn. Brain Res.* 25 963–981. 10.1016/j.cogbrainres.2005.09.021 16307871

[B23] Myrberg S. Riad T. (2015). The prosodic hierarchy of Swedish. *Nordic J. Linguist.* 38 115–147. 10.1080/13682820410001654874 15204444

[B24] Norris D. McQueen J. M. (2008). Shortlist B: A Bayesian model of continuous speech recognition. *Psychol. Rev.* 115 357–395. 10.1037/0033-295X.115.2.357 18426294

[B25] Novén M. (2021). *Brain Anatomical Correlates of Perceptual Phonological Proficiency and Language Learning Aptitude.* (Ph.D.thesis). Lund: Lund University.

[B26] Novén M. Schremm A. Horne M. Roll M. (2021). Cortical thickness of left anterior temporal areas affect processing of phonological cues in native speakers. *Brain Res.* 1750:147150. 10.1016/j.brainres.2020.147150 33039411

[B27] Öhman S. (1967). Word and sentence intonation: a quantitative model. *STL-QPSR* 8 20–54.

[B28] Osterhout L. Holcomb P. J. (1992). Event-related brain potentials elicited by syntactic anomaly. *J. Mem. Lang.* 31 785–806. 10.1016/0749-596X(92)90039-Z

[B29] Pinker S. (1991). Rules of language. *Science* 253 530–535. 10.1126/science.185798 1857983

[B30] Riad T. (1992). *Structures in Germanic Prosody.* (Ph.D.thesis). Stockholm: Stockholm University.

[B31] Riad T. (1998). The origin of Scandinavian tone accents. *Diachronica* 15 63–98. 10.1075/dia.15.1.04ria 33486653

[B32] Riad T. (2012). Culminativity, stress and tone accent in Central Swedish. *Lingua* 122 1352–1379. 10.1016/j.lingua.2012.07.001

[B33] Riad T. (2014). *The Phonology of Swedish.* Oxford: Oxford University Press.

[B34] Riad T. (2015). *Prosodin i Svenskans Morfologi.* Stockholm: Morfem förlag.

[B35] Rischel J. (1963). Morphemic tone and word tone in Eastern Norwegian. *Phonetica* 10 154–164. 10.1159/000258166

[B36] Rodriguez-Fornells A. Clahsen H. Lleó C. Zaake W. Münte T. F. (2001). Event-related brain responses to morphological violations in Catalan. *Cogn. Brain Res.* 11 47–58. 10.1016/S0926-6410(00)00063-X 11240111

[B37] Roll M. (2015). A neurolinguistic study of South Swedish word accents: electrical brain potentials in nouns and verbs. *Nordic J. Linguist.* 38 149–162. 10.1017/S0332586515000189

[B38] Roll M. Horne M. Lindgren M. (2010). Word accents and morphology—ERPs of Swedish word processing. *Brain Res.* 1330 114–123. 10.1016/j.brainres.2010.03.020 20298679

[B39] Roll M. Söderström P. Frid J. Mannfolk P. Horne M. (2017). Forehearing words: Pre-activation of word endings at word onset. *Neurosci. Lett.* 658 57–61. 10.1016/j.neulet.2017.08.030 28823890

[B40] Roll M. Söderström P. Horne M. (2013). Word-stem tones cue suffixes in the brain. *Brain Res.* 1520 116–120. 10.1016/j.brainres.2013.05.013 23685193

[B41] Roll M. Söderström P. Mannfolk P. Shtyrov Y. Johansson M. van Westen D. (2015). Word tones cueing morphosyntactic structure: neuroanatomical substrates and activation time course assessed by EEG and fMRI. *Brain Lang.* 150 14–21. 10.1016/j.bandl.2015.07.009 26291769

[B42] Schremm A. Novén M. Horne M. Roll M. (2019). Brain responses to morphologically complex verbs: an electrophysiological study of Swedish regular and irregular past tense forms. *J. Neurolinguistics* 51 76–83. 10.1016/j.jneuroling.2019.01.006

[B43] Schremm A. Noveìn M. Horne M. Söderström P. van Westen D. Roll M. (2018). Cortical thickness of planum temporale and pars opercularis in native language tone processing. *Brain Lang.* 176 42–47. 10.1016/j.bandl.2017.12.001 29223785

[B44] Söderström P. Horne M. Frid J. Roll M. (2016). Pre-activation negativity (PrAN) in brain potentials to unfolding words. *Front. Hum. Neurosci.* 10:512. 10.3389/fnhum.2016.00512 27777558 PMC5056166

[B45] Söderström P. Horne M. Roll M. (2017). Stem tones pre-activate suffixes in the brain. *J. Psycholing. Res.* 46 271–280. 10.1007/s10936-016-9434-2 27240896 PMC5368231

[B46] Söderström P. Roll M. Horne M. (2012). Processing morphologically conditioned word accents. *Ment. Lex.* 7 77–89. 10.1075/ml.7.1.04soe 33486653

[B47] Trubetzkoy N. (1958). *Grundzüge der Phonologie.* Göttingen: Vandenhoeck & Ruprecht.

[B48] Ullman M. T. Corkin S. Coppola M. Hickok G. Growdon J. H. Koroshetz W. J. (1997). A neural dissociation within language: evidence that the mental dictionary is part of declarative memory, and that grammatical rules are processed by the procedural system. *J. Cogn. Neurosci.* 9 266–276. 10.1162/jocn.1997.9.2.266 23962016

[B49] Zora H. Riad T. Ylinen S. (2019). Prosodically controlled derivations in the mental lexicon. *J. Neuroling.* 52:100856. 10.1016/j.jneuroling.2019.100856

